# Intracellular iron sensing by the direct binding of iron to regulators

**DOI:** 10.3389/fpls.2015.00155

**Published:** 2015-03-11

**Authors:** Takanori Kobayashi, Naoko K. Nishizawa

**Affiliations:** ^1^Japan Science and Technology Agency, PRESTOKawaguchi, Japan; ^2^Research Institute for Bioresources and Biotechnology, Ishikawa Prefectural UniversityNonoichi, Japan

**Keywords:** iron deficiency, IDEF1, HRZs/BTS, sensor, signaling

## Introduction

Iron (Fe) is an essential micronutrient for virtually all living organisms; it is incorporated into numerous proteins as Fe-sulfur (S) clusters, heme, or free Fe, and it mediates many metabolic processes, including photosynthesis and cellular respiration. Due to the low solubility and high reactivity of Fe, living cells must tightly regulate Fe acquisition in response to changes in Fe availability in the environment. The molecular components involved in Fe acquisition and its regulation have been studied extensively in plants, animals, fungi, and bacteria, revealing specific mechanisms in each kingdom. Fe acquisition in higher plants is mediated by a chelation-based pathway using mugineic acid family phytosiderophores (Strategy II) or a reduction-based pathway using ferric-chelate reductases (Strategy I), depending on the species (Römheld and Marschner, 1986). Molecular components involved in both pathways are induced under Fe deficiency and repressed under Fe sufficiency, primarily at the transcript level; however, protein-level regulation has also been reported for some components (Lan et al., 2011; Kobayashi and Nishizawa, 2012). To achieve expressional regulation based on Fe availability, Fe sensing systems must exist, but the signals and sensors have not been clarified in plants. Although various metabolites affected by Fe availability are thought to influence Fe deficiency responses, we propose that the direct binding of Fe to expressional regulators is the primary Fe sensing event in plant cells, similar to the mechanisms in animals, fungi, and bacteria. The recent identification of the Fe-binding regulators IDEF1 and HRZs/BTS in plants supports this hypothesis.

## Intracellular Fe sensing in animals, fungi, and bacteria

In animals, Fe homeostasis is mediated by complicated transcriptional and post-transcriptional regulatory networks. Among them, the Fe regulatory protein (IRP)/Fe-responsive element (IRE) system plays a principal role in the cellular response to Fe (Anderson et al., 2012). Under Fe deficiency IRP1 and IRP2 bind to IREs, stem-loop structures present in various mRNAs that are involved in Fe homeostasis. IRP binding to an IRE increases the stability of mRNAs for Fe acquisition such as transferrin receptor, and inhibits the translation of proteins for Fe sequestration such as ferritin. Under Fe-sufficient conditions, IRP-IRE binding becomes less dominant because of two Fe-sensing mechanisms. First, an Fe-S cluster binds to IRP1 and converts it to cytosolic aconitase, inhibiting its mRNA binding activity. Second, IRP2 is ubiquitinated and subjected to proteasomal degradation via the function of F-box leucine-rich repeat protein 5 (FBXL5). Fe binding to FBXL5 triggers IRP2 degradation, while the Fe-free form of FBXL5 is rapidly degraded. These two mechanisms ensure preferential IRP-IRE binding under Fe deficiency. Animal cells possess additional Fe-sensing mechanisms, including abolishment of the hypoxia-inducible factor (HIF) regulatory pathway by Fe-requiring HIF prolyl hydroxylases (Thompson and Bruick, 2012) and de-repression of Bach-mediated transcriptional regulation by heme-Bach binding (Igarashi and Watanabe-Matsui, 2014).

In *Saccharomyces cerevisiae*, the transcription factors Aft1 and Aft2 are major players in the Fe deficiency response. Aft1/2 nuclear function is inhibited by an Fe-replete signal generated in mitochondria via Fe-S clusters, the cytosolic monothiol glutaredoxins Grx3 and Grx4, and the BolA-like protein Fra2. These proteins and Fe-S clusters form a cytosolic complex that enters the nucleus and dissociates Aft1 and Aft2 from their target *cis*-acting elements (Ueta et al., 2012; Poor et al., 2014). Thus, Fe-S clusters are primary signaling molecules for Fe deficiency responses in yeast cells.

Bacteria sense Fe by binding directly to members of the ferric uptake regulator (Fur) family (Lee and Helmann, 2007). Fur from *Escherichia coli* is a transcriptional repressor that binds to *cis*-acting elements and ferrous Fe. When cellular Fe is scarce, Fur does not bind to Fe; this decreases the affinity between Fur and its *cis*-acting elements, releasing the repression of Fe uptake-related genes.

Despite the diverse molecular mechanisms for Fe acquisition and regulation across kingdoms, all of the above-mentioned regulatory events are mediated by the direct binding of Fe or Fe-containing prosthetic groups (Fe-S clusters and heme) to regulatory proteins. Such direct Fe sensing is thought to be advantageous in rapidity and accuracy compared with indirect Fe sensing through metabolic changes, though the latter would also be important for fine-tuning the response.

## Fe-binding regulators in plants

**Figure 1:**
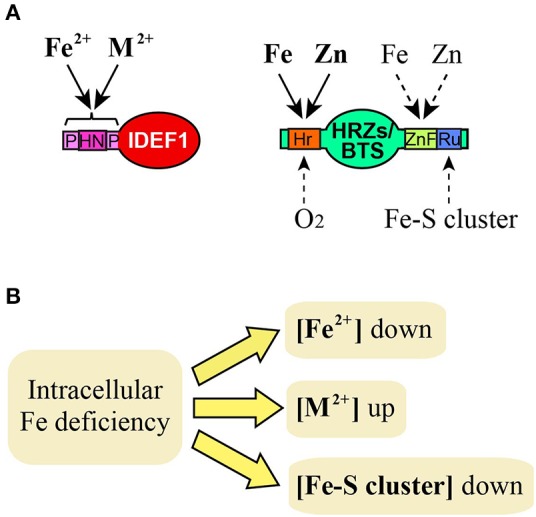
**Possible Fe signals recognized by IDEF1 and HRZs/BTS. (A)** Known or putative binding of Fe and related molecules to domains of IDEF1 and HRZs/BTS. Solid and broken lines indicate known and putative bindings, respectively. M^2+^, divalent metal ions such as Zn, copper and nickel. HN, histidine-asparagine repeat; P, proline-rich region; Hr, hemerythrin domains; ZnF, Zn finger domains; Ru, rubredoxin-type fold. **(B)** Deduced effects of intracellular Fe deficiency on concentrations of Fe and related molecules which may act as Fe signals.

Two types of Fe-binding regulators for Fe deficiency responses are known in higher plants: IDEF1 and HRZs/BTS (Figure 1A). IDEF1 is a Gramineae-specific transcription factor that positively regulates various genes involved in Fe uptake and translocation (Kobayashi et al., 2007, 2009). Analyses using transgenic rice plants revealed that IDEF1 is especially important for the early response to Fe deficiency. Nevertheless, *IDEF1* transcription is not induced in response to Fe deficiency, suggesting that IDEF1 regulates Fe deficiency responses by receiving a signal. IDEF1 possesses characteristic histidine-asparagine repeats and proline-rich regions that bind ferrous Fe and other divalent metals reversibly (Figure 1A; Kobayashi et al., 2012). These metal-binding regions are essential for overexpressed IDEF1 to enhance Fe deficiency responses at the early stages of Fe deficiency, although a direct relationship between metal binding and IDEF1 function has not been proven.

The second group of Fe-binding regulators in plants is HRZs/BTS; its members have been designated OsHRZ1 and OsHRZ2 in rice (Kobayashi et al., 2013) and BTS in *Arabidopsis thaliana* (Long et al., 2010). *OsHRZ1, OsHRZ2*, and *BTS* are transcriptionally induced under Fe deficiency, and their homologs are widely conserved among plants and algae (Urzica et al., 2012; Kobayashi et al., 2013). HRZs/BTS contain hemerythrin domains, three zinc (Zn) finger domains, including a RING finger, and a rubredoxin-type fold in each protein (Figure 1A). The hemerythrin domain binds ferrous Fe and molecular oxygen in animals and bacteria (Stenkamp, 1994), and is present in human FBXL5 as an Fe-binding and -sensing domain (Salahudeen et al., 2009; Vashisht et al., 2009; Thompson et al., 2012). *In vitro* studies revealed that the N-terminal regions of OsHRZ1, OsHRZ2, and BTS containing hemerythrin domains bind Fe as well as Zn (Kobayashi et al., 2013; Selote et al., 2015). OsHRZ1, OsHRZ2, and BTS are negative regulators of Fe deficiency responses; their knockdown results in enhanced tolerance to Fe deficiency and Fe accumulation in rice and *Arabidopsis* (Long et al., 2010; Kobayashi et al., 2013; Selote et al., 2015). OsHRZ1, OsHRZ2, and BTS also possess ubiquitination activity, which is likely attributed to the RING finger domain (Kobayashi et al., 2013). Selote et al. (2015) demonstrated that BTS binds and destabilizes two *Arabidopsis* basic helix-loop-helix (bHLH) proteins, ILR3 and AtbHLH115, most likely through proteasome-mediated degradation following ubiquitination. ILR3 and AtbHLH115 bind to another *Arabidopsis* bHLH protein, PYE, which regulates various genes involved in Fe homeostasis (Long et al., 2010). ILR3 regulates the expression of the *Arabidopsis* vacuolar Fe transporter-like genes *AtVTL1, AtVTL2*, and *AtVTL5*, which are thought to sequester Fe into vacuoles under Fe-replete conditions (Rampey et al., 2006; Gollhofer et al., 2014). These results suggest that BTS regulates Fe homeostasis via the ubiquitination of ILR3 and AtbHLH115, and modulation of the PYE-mediated Fe deficiency response; however, the precise functions of ILR3 and AtbHLH115 in Fe homeostasis are unclear. The ubiquitination targets of rice HRZs have not been identified, although bHLH proteins similar to PYE, ILR3, and AtbHLH115 are present in rice.

A common property of IDEF1 and HRZs/BTS is their ability to bind non-Fe metals, including Zn, which compete with Fe for binding (Figure 1A; Kobayashi et al., 2012, 2013). This suggests that Fe sensing in plant cells is mediated either by Fe itself, non-Fe metals, or both. Non-Fe metals might act agonistically or antagonistically. The former case has been demonstrated for bacterial Fur, which is able to bind manganese in place of Fe, acting similarly to the Fe-bound form (Lee and Helmann, 2007). However, the antagonistic function of non-Fe metals might be more plausible in plant cells because the concentrations of non-Fe metals in plant tissues increase during Fe deficiency (Figure 1B). Recently, dynamic imaging by FRET sensors enabled an estimation of cytosolic concentration of free Zn^2+^ in *Arabidopsis* roots (Lanquar et al., 2014). However, the concentrations of free Fe remain unknown in plant cells. Monitoring the free concentrations of Fe and other metals in plant cells under various nutrient conditions would be important for clarifying Fe sensing by these metals. Interestingly, the subcellular localization of the *Arabidopsis* transporter IRT1, which is involved in the uptake of ferrous Fe and other divalent metals by roots, is regulated by non-Fe metals, but not by Fe (Barberon et al., 2014). IRT1 shuttles between the plasma membrane and early endosome/trans-Golgi network dependent on monoubiquitination; however, the depletion of non-Fe metals stabilizes IRT1 localization at the root surface. This regulation is thought to be a protective mechanism to prevent excess uptake of non-Fe metals such as Zn and manganese (Thomine and Vert, 2013). This regulation also contributes to Fe homeostasis, thus it suggests the physiological suitability of non-Fe metals as a type of Fe signal.

Another suggested property of HRZs/BTS is sensing multiple Fe-related signals (Figure 1). In addition to binding Fe and Zn, the hemerythrin domains of HRZs/BTS may bind molecular oxygen, because these domains bind oxygen coordinated with Fe in invertebrates (Stenkamp, 1994). Human FBXL5 is destabilized under hypoxia as well as under Fe deficiency, suggesting tight linkage between Fe deficiency responses and cellular redox status (Salahudeen et al., 2009; Vashisht et al., 2009; Thompson et al., 2012). HRZs/BTS possess additional domains that are potentially involved in Fe sensing, including Zn fingers and rubredoxin-type folds. The coordination of Zn to Zn fingers usually contributes to protein structure, but some Zn fingers are known to function as Zn sensors (Bird et al., 2003). Fe also binds to some Zn fingers (Conte et al., 1996; Besold et al., 2010). Rubredoxin-type folds in bacteria bind Fe-S clusters, which are important Fe signaling molecules in animals and yeast. These observations suggest that HRZs/BTS sense and coordinate more than one type of Fe signal. Although the mechanisms underlying the regulation of Fe homeostasis are thought to be distinct between plants and other organisms, the identification of HRZs/BTS suggests the partial conservation of Fe-sensing mechanisms across kingdoms.

## Identifying direct Fe sensors in plants

Recently, we proposed that a cellular Fe sensor must meet the following three criteria: (i) bind to Fe or intimately related molecule(s), (ii) change its function in response to binding, and (iii) regulate Fe homeostasis (Kobayashi and Nishizawa, 2014). Both IDEF1 and HRZs/BTS bind Fe and Zn, and they regulate Fe deficiency responses, satisfying criteria (i) and (iii), but there is no clear evidence to support criterion (ii). Very recently, Selote et al. (2015) reported that BTS stability is dependent on Fe, providing evidence linked to criterion (ii). They showed that BTS produced *in vitro* using a wheat germ extract system was less abundant when Fe was included in the translation reaction mixture. This effect was abolished by a point mutation at a putative Fe-binding residue in the hemerythrin domain, suggesting that Fe binding to the hemerythrin domain destabilizes and inhibits the function of BTS. When expressed *in planta*, deletion of the hemerythrin domains from BTS resulted in increased protein stability. However, complementation tests using an *Arabidopsis bts* knockdown mutant revealed that a truncated version of BTS without the hemerythrin domains functioned more or less similarly to full-length BTS with respect to physiological Fe deficiency responses (Selote et al., 2015), suggesting the limited importance of Fe-mediated BTS degradation via the hemerythrin domains. Further characterization of the mechanisms underlying the degradation and function of HRZs/BTS in relation to the binding of Fe, Zn, oxygen, or Fe-S clusters is essential to identify whether these regulators are central Fe sensors that determine downstream responses to Fe deficiency. Similarly, identification of the molecular mechanism by which IDEF1 alters its function due to Fe or Zn binding is needed. It is possible that primary Fe sensing in plant cells is established by unknown factors, while IDEF1 and HRZs/BTS modulate these events and mediate appropriate Fe deficiency responses. In this scenario, the identification of a central regulator that changes its function in response to binding Fe or an intimately related molecule would be key to verifying it as a central Fe sensor.

### Conflict of interest statement

The authors declare that the research was conducted in the absence of any commercial or financial relationships that could be construed as a potential conflict of interest.
